# Post-traumatic exogenous endophthalmitis caused by *Nocardia farcinica*

**DOI:** 10.1186/s12348-021-00245-3

**Published:** 2021-06-01

**Authors:** Marie Česká Burdová, Kateřina Donátová, Gabriela Mahelková, Vanda Chrenková, Dagmar Dotřelová

**Affiliations:** 1 Department of Ophthalmology, Charles University, 2nd Faculty of Medicine and University Hospital in Motol, Prague, Czech Republic; 2 Department of Physiology, Charles University, 2nd Faculty of Medicine, Prague, Czech Republic; 3 Department of Medical Microbiology, Charles University, 2nd Faculty of Medicine and University Hospital in Motol, Prague, Czech Republic

**Keywords:** *Nocardia farcinica*, Post-traumatic endophthalmitis

## Abstract

A case report of post-traumatic exogenous endophthalmitis caused by *Nocardia farcinica,* including treatment procedures, microbiology examination, and systemic medications. A 23-year-old male suffered a penetrating corneal injury that was treated with sutures. On the thirteenth day after the final suture was removed, an anterior uveitis developed and progressed to whitish, plump, nodular, and tufted exudates within the anterior chamber over the next 10 days; this led to an indication for intraocular surgery. Anterior chamber lavage and resection of solid fibrinous exudates (using a vitrectomy knife) for a complete microbiological examination were performed. *Nocardia farcinica* was identified. Systemic medications were chosen according to sensitivity, and a fixed combination of sulfamethoxazole 400 mg/trimethoprim 80 mg was administered long-term (months). In this case, accurate, early detection of an atypical infectious agent and determination of its sensitivity to antibiotic treatment enabled effective treatment that achieved the best functional and anatomical results under the circumstances.

## Introduction

Endophthalmitis is a bacterial or fungal intraocular inflammation that infiltrates the vitreous humor and/or the anterior chamber of the eye [1]. In most cases, endophthalmitis is exogenous. Endophthalmitis is an acute or subacute disease, if not appropriately treated. Among patients with infectious endophthalmitis, post-traumatic endophthalmitis (PE) comprises ∼ 25–31% of cases [2]. The reported incidence rate of endophthalmitis following open-globe injury ranges from 0 to 16.5% [3], although early surgical repair and prophylactic systemic antibiotics can reduce this incidence to < 1% [4].

Nocardia species can also lead to PE. They are aerobic, gram-positive, filamentous, branching bacteria that are ubiquitous in the environment [5, 6]. They are found everywhere, from sludge and soil to water contaminated with soil, deep-sea sediments, and desert habitats. They can be cultivated from dust inside of dwellings and pools of natural water [6–8].

In addition to PE, Nocardia species can cause human skin, lungs, and central nervous system infections, as well as systemic nocardiosis, especially in immunocompromised patients [5–7]. In terms of ocular pathology, Nocardia can cause keratitis, keratoconjunctivitis*,* scleritis, dacryocystitis, orbital cellulitis, and both exogenous and endogenous endophthalmitis have been described [9–13].

This paper reports on a case of PE caused by *Nocardia farcinica* after a penetrating corneal injury and its treatment.

## Case report

A 23-year-old healthy immunocompetent, Caucasian male was admitted to our hospital with a penetrating corneal injury caused by a razor blade; he was removing a vignette from the windshield of his car using the razor blade.

The corneal wound was just below the center of the cornea and punctured the anterior lens capsule. Corneal surgery was performed 7 h after the injury.

To prevent PE, during the pre and postoperative period, the patient was treated with a combination of intravenous vancomycin and ceftazidime, followed by oral cefuroxime, with the topical levofloxacin. The postoperative course was without complications. The individual knotted sutures were removed separately during the 4th and 5th months after surgery. The patient came back with mild non-granulomatous anterior uveitis with fibrin in the anterior chamber on the thirteenth day after the removal of the last corneal suture. Given the slow progression of clinical signs and symptoms, phacogenic uveitis or bacterial/fungal endophthalmitis were considered.

Therapy was initiated with topical mydriatic drops, topical and subconjunctival dexamethasone, followed by three boluses of intravenous methylprednisolone. Systemic therapy consisted of combined antibiotics (cefuroxime 500 mg twice a day and clindamycin 300 mg every 6 h) and an antimycotic (fluconazole 400 mg twice a day).

**Fig. 1:**
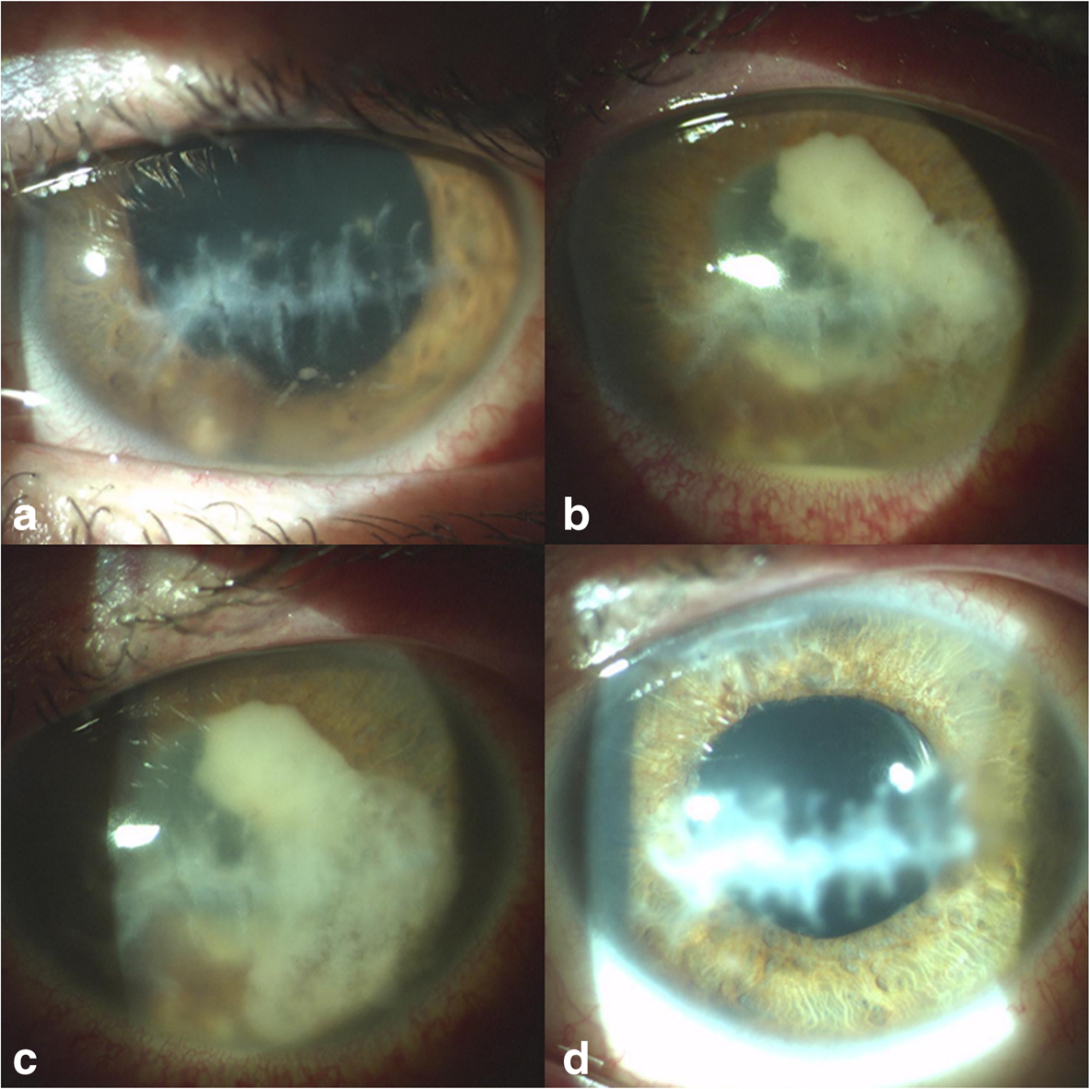
Post-traumatic corneal scar. Mild non-granulomatous anterior uveitis with fibrin in the anterior chamber. The nodular exudates grew anteriorly towards the corneal endothelium and imitated an iris cyst (**a**). A hypopyon in the anterior chamber and white, plump, fluffy hemispherical exudates progressing into the anterior chamber (**b**). Whitish, plump, nodular, and tufted exudates continued to develop within the anterior chamber (**c**). The eye 5 years after cataract surgery. The eye is completely without signs and symptoms of inflammation and the patient is without any local and systemic medications (**d**)

After a transitory improvement, the clinical signs continued to progress during the topical and systematic therapy. Gradually, fluffy (soft) exudates appeared on the endothelium along with the formation of bounded nodular exudates on the anterior surface of the lens and in the inferior temporal quadrant of the pupillary border. The nodular exudates grew anteriorly towards the corneal endothelium and imitated an iris cyst (Fig. 1a). A hypopyon appeared in the anterior chamber, and white, plump, and fluffy hemispherical exudates erupted into the anterior chamber and dispersed therein (Fig. 1b).

Over the next 10 days, whitish, plump, nodular, and tufted exudates continued to develop within the anterior chamber. Eventually, the anterior chamber was completely filled with the material (Fig. 1c), and significant secondary glaucoma developed. Repeated ultrasonography of the eye showed a normal appearance of the vitreous body The results of serological tests were negative.

Based on this evolution, intraocular surgery was indicated. The collection of exudates plus iris tissue, anterior chamber lavage, synechiolysis, basal iridectomy, and resection of the solid fibrinous exudates (using a vitrectomy knife) were collected for a complete microbiological examination. Lastly, cefuroxime was applied to the anterior chamber.

**Fig. 2:**
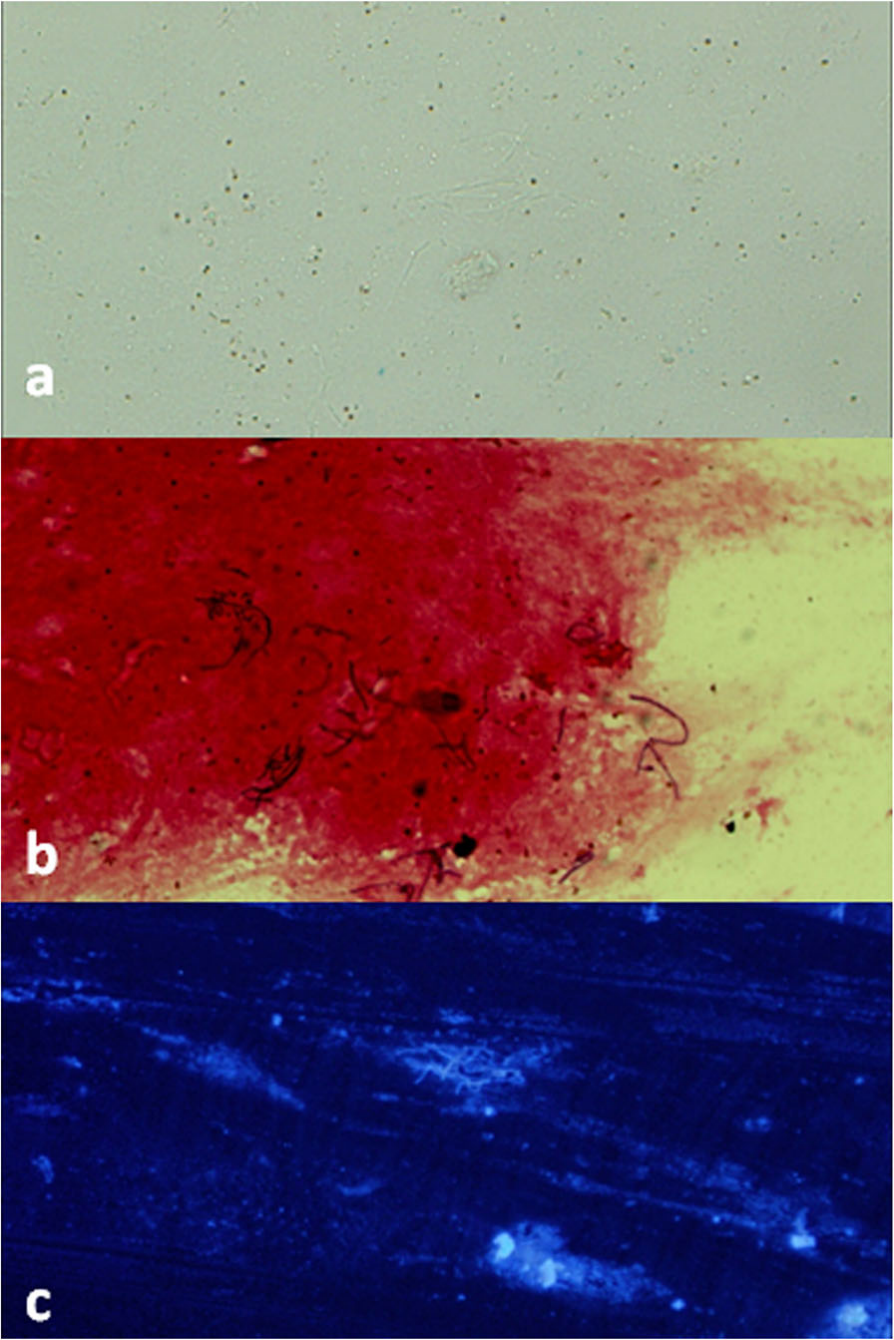
Microscopy using white light source (**a**), Gram stain (**b**), and Calcofluor white staining (**c**) of Nocardia filaments

Gram-positive filaments, which were suggestive of Nocardia or Streptomyces, were seen in the perioperatively obtained sample. Fluorescence microscopy showed slender filaments, probably Actinomycosis or Nocardia (Fig. 2). Subsequently, the sample was specified as *Nocardia farcinica*. The antibiotic sensitivity of the cultivated *Nocardia farcinica* is shown in Table 1. Postoperative treatment included the topical antibiotics ofloxacin and sulphacetamide10%. The systemic medication was chosen according to sensitivity results, and a fixed combination of sulfamethoxazole/trimethoprim (i.e., 400 mg/80 mg) in the form of Biseptol® 480 mg (Polfa S.A.) was administered as two capsules every 8 h.

**Table 1:** Antibiotic qualitative and quantitative (mg/l) sensitivity of *Nocardia farcinica*; (S - Antibiotic sensitive, R - Antibiotic-resistant, I - Antibiotic intermediate sensitivity)

Antibiotics	Sensitivity	mg/l	Antibiotics	Sensitivity	mg/l	Antibiotics	Sensitivity	mg/l
Trimethoprim/sulfonamide	S	2	Linezolid	S	2	Ciprofloxacin	R	4
Imipenem	S	4	Moxifloxacin	I	2	Cefepime	R	32
Cefoxitin	R	128	Amoxicillin/acid clavulanic	I	16	Amikacin	S	1
Ceftriaxone	R	64	Doxycycline	I	4	Minocycline	I	4
Tigecycline	R	4	Tobramycin	R	16	Clarithromycin	R	16

After consultation with the Department of Medical Microbiology, long-term therapy lasting for several months with a fixed combination of sulfamethoxazole/trimethoprim (400 mg/80 mg) was recommended. Eye irritation and mild anterior uveitis relapses were recorded and resulted in temporary discontinuation of systemic antibiotic therapy. Surgery for the complicated cataract and implantation of a monofocal intraocular lens was indicated 10 months after the PE. During cataract surgery, a sample of aqueous humour was removed for microbiological examination, and a negative result was confirmed. The pre and postoperative period was covered by the topical antibiotic amikacin 5 mg/ml, sulfacetamide 10%, and levofloxacin. The treatment with the systemic antibiotics sulfamethoxazole/trimethoprim (400 mg/80 mg) was discontinued definitely 1 month after the cataract surgery, based on the negative result from microbiology. In total, systemic treatment lasted 12 months. The final decimal value for the best-corected visual acuity is 0.5 (Fig. 1d).

## Discussion

From the ophthalmologic point of view, PE caused by a Nocardia species is extremely rare, even though Nocardia species are ubiquitous. In cases caused by very uncommon pathogens, the proper diagnosis and causal treatment of PE can be exceedingly difficult. An incorrect diagnosis is often assumed, and a more common pathogen of mycotic origin is then considered. Because of the delayed causal treatment in these types of cases, significant ocular morbidity, including enucleation of the eye, cannot be prevented in most cases [14, 15].

Very few cases of PE caused by Nocardia species, after a penetrating eye injury, have ever been described in the literature, and to the best of our knowledge, none were caused by *Nocardia farcinica*; and common injury mechanisms included penetration by a fragment of a windshield, a palm leaf, or a plastic hose [16–18]. All suggest that Nocardia can apparently grow on smooth surfaces, which fits with one explanation of our patient’s infection, i.e., the initial injury being caused by a razor blade slipping on the car windshield.

As with other infections, immunological status also plays a crucial role in Nocardiosis [19]. This was confirmed by an extensive retrospective study of the relationship between manifestations and outcomes of Nocardia infections relative to the immunocompetence of patients. Of the at least 92 Nocardia species [20], the most common infectious agents were found to be *Nocardia asteroids* (73%), *Nocardia farcinica* (9%), and *Nocardia brasiliensis* (4%). The majority of patients (60%) were immunosuppressed. No cases of PE were described in patients without immune impairment [5]; however, it is noteworthy that our patient was immunocompetent.

The mechanism and exact time of the infection in our patient is not clear. During the 6 months post-injury period, the eye was calm. There were no signs of post-traumatic irritation of the eye, the corneal wound healed and no signs of inflammation were ever noted**.**

There are two possible explanations for the intraocular penetration of *Nocardia farcinica*. Our first potential explanation is that the infectious agent penetrated the eye during the primary injury and was encapsulated there, possibly around the injury to the anterior lens capsule. If so, the interval between injury and PE would be 6 months.

Compte et al. described a case in which the interval between an eye injury caused by a palm tree leaflet and the PE was 2 months. In addition to broad-spectrum antibiotics, the patient was also treated with glucocorticoids during the post-traumatic period, which could have prolonged the interval between the injury and PE. *Nocardia kruczakiae* was determined as the agent [17].

Rodriquez-Lozano et al. also described a long interval between the time of injury and the onset of PE. In their patient, a perforating keratoplasty was performed 5 months after the primary injury, and PE caused by *Nocardia nova* developed 1 year after the injury. Whether the PE was a consequence of the primary injury or the surgery remained unclear [18].

Our second potential explanation is that the infectious agent entered the eye at the time of the last corneal sutures removal. If so, this suture was not established intrastromally during the primary suture on the hypotonic eye but instead was guided through the entire thickness of the cornea and into the anterior chamber.

We think the second explanation is more likely. After verification of the pathogen, the patient was reinterviewed and stated that he repeatedly swam in a natural pond, both before and after the corneal sutures had been removed. Since it is generally accepted that Nocardia is ubiquitous pathogens that can also be present in reservoirs and pools of natural water [6–8]**,** we assume that *Nocardia farcinica* adhered to the sutures during swimming and the pathogen was inoculated directly into the anterior chamber during suture extraction. This hypothesis is supported by the fact that no signs of keratitis were found at the time of the anterior uveitis occurrence.

In Nocardia infections, the posterior segment is initially normal or only slightly involved. However, a large proportion of patients (75–83%) show nodules on the corneal endothelium or on the iris. The anterior smooth surface of the lens, in the lower periphery of the posterior chamber, is probably an optimal place for Nocardia species to grow [14]. This agrees with our experience. Initially, nodular exudates began to spread on the corneal endothelium and on the surface of the lens and iris and then spread to the anterior chamber. The posterior segment was also normal.

Nocardia species cultivation is complicated. Hudson et al. described a case of PE with similar manifestations as in our patient. Even a diagnostic pars plana vitrectomy and sectoral iridectomy were performed, with the culture results of the aspirated material being negative. Ultimately, enucleation of the eye was performed, and *Nocardia asteroides* was found [16].

The success of the diagnosis and subsequent treatment of PE caused by Nocardia species is always based on interdisciplinary cooperation and collaboration with the Department of Medical Microbiology [18]. Consultations on optimal sample collection and the transport of pathological material, as well as proper testing procedures, are crucial. Accurate, early detection of the infectious agent and administration of maximally effective treatments is crucial for obtaining optimal functional and anatomical results. However, our case demonstrates that even when the course and resolution are not straightforward, the final outcome and visual acuity can be very satisfactory.

## Data Availability

Data sharing is not applicable to this article as no datasets were generated or analyzed during the current study.

## Abbreviation

PE: Post-traumatic endophthalmitis
